# The relationship between apelin and cardiac parameters in patients on peritoneal dialysis: is there a new cardiac marker?

**DOI:** 10.1186/1471-2369-15-18

**Published:** 2014-01-16

**Authors:** Serhat Karadag, Savas Ozturk, Meltem Gursu, Ahmet Gurdal, Filiz Basinoglu, Servet Yigit, Zeki Aydin, Sami Uzun, Abdullah Sumnu, Huseyin Oflaz, Rumeyza Kazancioglu

**Affiliations:** 1 Department of Nephrology, Haseki Training and Research Hospital, Adivar Caddesi, Aksaray, Fatih, Istanbul, Turkey; 2 Istanbul Medical Faculty, Department of Cardiology, Istanbul University, Istanbul, Turkey; 3 Department of Biochemistry, Haseki Training and Research Hospital, Istanbul, Turkey; 4 Medical Faculty, Department of Nephrology, Bezmialem Vakif University, Istanbul, Turkey

**Keywords:** Apelin, Echocardiography, Hypervolemia, Peritoneal dialysis

## Abstract

**Background:**

Many markers have been proposed for CVD risk assessment in dialysis population. Apelin is a peptide that has roles in cardiovascular functions and volume regulation namely vasodilation, decreased blood pressure (BP), positive inotropic effect and inhibition of antidiuretic hormone release. The aim of this study was to examine relationship of apelin levels with echocardiographic findings and laboratory parameters related with cardiovascular function and bone mineral metabolism among peritoneal dialysis (PD) patients.

**Methods:**

This is a cross-sectional study in which chronic PD patients aged between 18 and 80 without active cardiac, infectious or malignant diseases and hypervolemia have been included. Apelin-36 levels and echocardiographic findings were recorded as well as clinical and laboratory data.

**Results:**

Of the 53 patients, the mean age and female/male ratio was 52.8 ± 15.3 years and 30/23, respectively. Mean apelin level was 1.45 ± 0.37 ng/ml. Gender, drugs (renin-angiotensin-aldosteron inhibitors, statins), presence of left ventricular hypertrophy, diabetes mellitus, hypertension, hyperlipidemia and significant residual renal function did not affect apelin-36 levels. Apelin-36 was correlated negatively with age and left atrium diameter; and positively with diastolic BP, ejection fraction (EF), total cholesterol, LDL-cholesterol, HDL-cholesterol, parathyroid hormone and alkaline phosphatase (ALP) levels. Diastolic BP, LDL-cholesterol, ALP and EF were found to be the independent determinants of apelin-36 levels with linear regression analysis.

**Conclusions:**

Apelinergic system has important roles in volume regulation, cardiovascular functions, lipid metabolism and bone mineral disorders in PD patients. Prospective studies with large population are required.

## Background

The mortality rate of patients with end stage renal disease (ESRD) is markedly elevated compared with the general population; and the major reason of this finding is cardiovascular disease (CVD) which is responsible for 40-60% of these deaths [1]. Left ventricular hypertrophy (LVH), that is more prevalent in peritoneal dialysis (PD) than hemodialysis (HD) population, is a strong indicator of cardiomyopathy and an important reason for sudden cardiac death [1-3]. About 40% of patients with ESRD are diagnosed to have congestive heart failure which results mostly from diastolic dysfunction and circulatory congestion [4]. Besides LVH, accelerated atherosclerosis, arteriosclerosis, and vascular remodeling are thought to cause increased CVD rate [1].

Peritoneal dialysis patients are different than HD patients in terms of CVD risk. About one third of PD patients are hypervolemic that is found to be related with increased mortality rate [5]. Moreover, traditional risk factors like hyperglycemia, hyperlipidemia and obesity are more prevalent in PD population [6].

Many markers have been proposed for CVD risk assessment in dialysis population. Apelin is a peptide of which the pathophysiological effects have been elucidated recently. It has been described in 1998 as the selective endogenous ligand of APJ receptor which is a G protein coupled membrane receptor [7,8]. APJ receptor has close homology with angiotensin-1 (AT-1) receptor although apelin and angiotensin do not share their receptors [9]. APJ receptors have been detected in endothelial cells of small intramyocardial, renal, pulmonary and bronchial vessels, coronary arteries, endocardial cells and vascular smooth muscle cells [10]. Preproapelin is located widely in human body mainly in central nervous system, placenta, kidneys, heart, lungs, adipose tissue and mammarian glands [11]. It has been claimed to be secreted by endothelial cells in conjunction with other vasoactive mediators [9].

Apelin is thought to play roles in cardiovascular functions and volume regulation like vasodilation and decreased blood pressure [12]; vasoconstriction in the presence of dysfunctional endothelium [13]; positive inotropic effects [14]; inhibition of antidiuretic hormone (ADH) release [15]; dilation of afferent and efferent arterioles, and vasoconstrictive effects on smooth muscle cells [16]. Apelinergic system is up regulated in early stages of heart failure possibly as a compensatory mechanism, and down regulated in later stages [17,18]. It is well known that bone mineral disorders and the related vascular calcification plays an important role in the pathogenesis of CVD in uremic patients. Studies have shown that apelin and its receptor, present in osteoblasts, suppress apoptosis and increase proliferation of osteoblasts [19,20]. Another study proposed that apelin is protective against vascular calcification through inhibition of osteoblastic differentiation of vascular smooth muscle cells [21].

The knowledge about the role of apelin in pathophysiology of cardiovascular disease is not sufficient enough in uremic patients, especially in PD patients. The aim of this study was to examine relationship of apelin-36 levels with echocardiographic findings and laboratory parameters which may be related with cardiovascular function and bone mineral metabolism among PD patients.

## Methods

Among 69 chronic PD patients followed in our PD unit; those aged between 18 and 80, with dialysis duration more than three months and without active cardiac (acute coronary syndrome, idiopathic dilated cardiomyopathy, infective endocarditis, decompensated heart failure, valvular heart disease, congenital heart diseases, atrial fibrillation and other arrythmias, pacemaker need, pericardial diseases), infectious or malignant disease and hypervolemia (clinically prominent dypnea, edema, pulmonary congestion findings, ascites, and cardiomegaly on radiograph) and those who gave informed consent have been included in the present study. Ethical approval was not gained due to the design of the study that does not necessitate invasive procedures or drug use.

Age, gender, body mass index (BMI), systolic, diastolic and mean blood pressures, primary kidney disease, dialysis duration, dialysis modality (CAPD-continuous ambulatory peritoneal dialysis-, CCPD-continuous cyclic peritoneal dialysis-, APD-automated peritoneal dialysis), Kt/V values, mean daily urine volume, residual glomerular filtration rates, residual and total creatinine clearances and all medications were recorded. The comorbidities of the patients including hypertension, hyperlipidemia, ischemic heart disease and diabetes mellitus were recorded. Pateints who were dignosed to have hypertension before, and those with blood pressure more than 140/90 mmHg in at least two measurements were regarded to have hypertension. Patients on antidiabetic treatment with previous diagnosis of diabetes mellitus; or those with fasting blood glucose above 126 mg/dl; or blood glucose level above 200 mg/dl at any time or blood glucose level above 200 mg/dl on second hour measurement of oral glucose tolerance test were recorded as diabetic. The diadnosis of hyperlipidemia was put according to the criteria of National Cholesterol Education Program-Adult Treatment Panel (NCEP-ATP) III regarding the age and other risk factors of the patients. Ischemic heart disease was diagnosed in patients with previous medical history (previous acute coronary syndrome, coronary artery by-pass surgery, history of baloon angioplasty or stent implantation, coronary lesions on coronary angiography, etc.), those with typical symptoms of coronary artery disease (angina pectoris or angina equivalents), patients with typical findings on electrocardiography, echocardiography, stress tests or coronary angiography.Blood samples for hematological and biochemical measurements were obtained after 12-hour fasting with their abdomen left empty the night before. Serum glucose, urea, creatinine, uric acid, total cholesterol, LDL-cholesterol, HDL-cholesterol, triglyceride, sodium, calcium, phosphorus, alkaline phosphatase (ALP), total protein, albumin, aspartate aminotransferase (AST) and alanine aminotransferase (ALT), parathyroid hormone (PTH), high sensitive C reactive protein (hsCRP), ferritin, hemoglobin and hematocrite levels were measured by appropriate methods.

### Measurement of apelin levels

Blood samples of 4 ml were drawn to tubes coated with ethylen diamin tetra acetic acid (EDTA). The samples were centrifuged at 1600 g for 15 minutes, and the plasma samples obtained were kept at -80°C for one month. The samples and the reactives were turned to room temperature prior to study. Plasma apelin-36 levels were studied by competitive enzyme immunoassay method using Phoenix Pharmaceuticals, Inc, Human Apelin-36 Enzyme Immunoassay kit (Range: 0-100 ng/ml). The concentration of the samples were calculated through calibration curves obtained from study of samples and standards with known levels.

General Electric VIVID-7 machine was used for echocardiographic examination by the same physician. Diameters of cardiac chambers were measured by M-mode ultrasonography. Ejection fraction (EF) was calculated by modified Simpson method. Left ventricular mass (LVM) was calculated by Devereux formula and left ventricular mass index (LVMI) was found by dividing LVM by BSA. Left ventricular hypertrophy was defined as LVMI above 110 gr/m^2^ and 134 gr/m^2^ for females and males, respectively [22].

Statistical analysis was conducted by SPSS (Statistical Package for Social Sciences) 15 for Windows standard version. Numerical values were expressed as mean ± standard deviation (SD). Intergroup comparisons were made by paired Student t-test and Mann Whitney U test when necessary. For nonnumerical parameters, for 2×2 contingency tables chi-square test and Fisher’s exact test when appropriate. Correlation analysis was conducted with Pearson test for parametric variables and Spearman’s rho correlation test for nonparametric variables. Linear regression analysis was performed by enter method with variables found to be related with apelin-36 levels in univariate analysis.

## Results

Fifty three patients were included in the study. Mean age and female/male ratio was 52.8 ± 15.3 years and 30/23, respectively. Dialysis modality was CAPD in 33 (62.3%), APD in 17 (32.1%) and CCPD in 3 (5.7%) patients. Demographic and clinical data are presented in Table 1. The most common etiology of ESRD was diabetes mellitus and the most common co-morbidity was hypertension (77%).

**Table 1:** Demographic and clinical data of the patients

		**Mean±SD**
**Age (years)**		52.8±15.3
**Female/male ratio**		30/23
**BMI (m**^**2**^**)**		27.7±6.5
**Systolic blood pressure (mmHg)**		128±21
**Diastolic blood pressure (mmHg)**		80±10
**Mean blood pressure (mmHg)**		96±14
**PD duration (months)**		41.7±24.9
**Primary kidney disease n (%)**	**Hypertension**	8 (15)
	**Unknown**	14 (26)
	**Diabetes mellitus**	14 (26)
	**Glomerulonephritis**	6 (11)
	**ADPKD**	3 (6)
	**Postrenal reasons**	8 (16)
**Co-morbidities n (%)**	**Hypertension**	41 (77)
	**Diabetes mellitus**	15 (17)
	**Hyperlipidemia**	21 (40)
	**Ischemic heart disease**	9 (28)

*BMI*; body mass index, *ADPKD*; autosomal dominant polycystic kidney disease.

The biochemical and hematological data are presented in Table 2. Mean plasma apelin-36 level was 1.45 ± 0.37 ng/ml. There was no difference between levels found in males and females (p = 0.15). Apelin-36 levels were similar in patients taking and not taking angiotensin converting enzyme inhibitors (p = 0.35), angiotensin receptor blockers (p = 0.31), renin angiotensin aldosteron system blockers in general (p = 0.84) and statins (p = 0.64).

**Table 2:** Biochemical and hematological parameters of the study group

	**Mean±SD**
**Glucose (mg/dl)**	136±82
**Urea (mg/dl)**	100±35
**Creatinine (mg/dl)**	8±2.9
**Uric acid (mg/dl)**	6±1.1
**Sodium (mmol/L)**	138±3.8
**Calcium (mg/dl)**	9.1±0.6
**Phosphorus (mg/dl)**	5±1.2
**Parathormon (pg/ml)**	560±429
**Total protein (g/dl)**	6.5±0.8
**Albumin (g/dl)**	3.8±0.4
**Total cholesterol (mg/dl)**	189±44
**HDL-cholesterol (mg/dl)**	42±17
**LDL-cholesterol (mg/dl)**	113±34
**Triglyceride (mg/dl)**	172±94
**AST (U/L)**	17±7
**ALT (U/L)**	17±11
**Alkaline phosphatase (U/L)**	135±193
**Hemoglobin (g/dl)**	10.8±1.4
**Hematocrite (%)**	32.7±4.1
**Ferritin (ng/ml)**	388±322
**hsCRP (mg/dl)**	2.0 ±4.3

*HDL*; high density lipoprotein, *LDL*; low density lipoprotein, *AST*; aspartate aminotransferase, *ALT*; alanine aminotransferase.

Echocardiographic findings are presented in Table 3. Patients with (n = 34) and without (n = 19) LVH had similar apelin-36 levels (1.41 ± 0.28 ng/ml vs. 1.53 ± 0.49 ng/ml, p = 0.29). Similarly apelin-36 levels were statistically not different in patients with or without ischemic heart disease (1.28 ± 0.29 ng/ml vs. 1.49 ± 0.38 ng/ml, p = 0.11). Presence of diabetes mellitus (1.52 ± 0.42 ng/ml vs. 1.43 ± 0.35 ng/ml; p > 0.05), hypertension (1.45 ± 0.51 ng/ml vs. 1.47 ± 0.15 ng/ml, p > 0.05) and hyperlipidemia (1.37 ± 0.77 ng/ml vs. 1.50 ± 0.38 ng/ml, p > 0.05) did not differ apelin-36 levels also.

**Table 3:** Echocardiographic findings of the study group

	**Mean±SD**
**Left atrium diameter (cm)**	3.58±0.56
**Left ventricle end diastolic diameter (cm)**	4.63±0.59
**Left ventricle end systolic diameter (cm)**	3.01±0.58
**Ejection fraction (%)**	62.06±8.9
**Interventricular septum thickness (cm)**	1.24±0.22
**Right ventricle diameter (cm)**	2.53±0.27
**Aorta diameter (cm)**	3.21±0.32
**Pulmonary artery diameter (cm)**	2.09±0.24
**Left ventricle mass (gram)**	246.8±82.6
**Left ventricle mass index (gr/m**^**2**^**)**	140.8±42.9

Mean urine volume and residual GFR of patients were 820 ± 790 ml and 3.4 ± 3.3 ml/min/1.73 m^2^, respectively. Patients with significant residual renal function meaning GFR ≥ 2 ml/min/1.73 m^2^ (n = 32) had similar apelin-36 levels with those without significant residual renal function (1.44 ± 0.38 ng/ml vs. 1.47 ± 0.36 ng/ml, p = 0.83). All patients met the criteria for dialysis adequacy with mean total, dialysate and renal Kt/V values of 2.51 ± 0.66, 1.80 ± 0.47 and 0.71 ± 0.71 respectively. The mean total creatinine clearance was 79.1 ± 29.2 ml/min/1.73 m^2^.

Among the demographic parameters, only age was negatively correlated with apelin-36 levels (r = -0.277, p = 0.044). Apelin-36 was positively correlated with diastolic blood pressure (r = 0.37, p = 0.006), total cholesterol (r = 0.271, p = 0.050), LDL-cholesterol (r = 0.313, p = 0.023), HDL-cholesterol (r = 0.317, p = 0.021), PTH (r = 0.322, p = 0.019) and ALP (r = 0.510, p <0.0001) levels. There was no correlation of apelin-36 with hematological variables, hsCRP, Kt/V, creatinine clearance and residual renal function. Among the echocardiographic parameters; apelin-36 was positively correlated with EF (r = 0.298, p = 0.030), and negatively with left atrium diameter (r = -0.288, p = 0.036).

**Figure 1:**
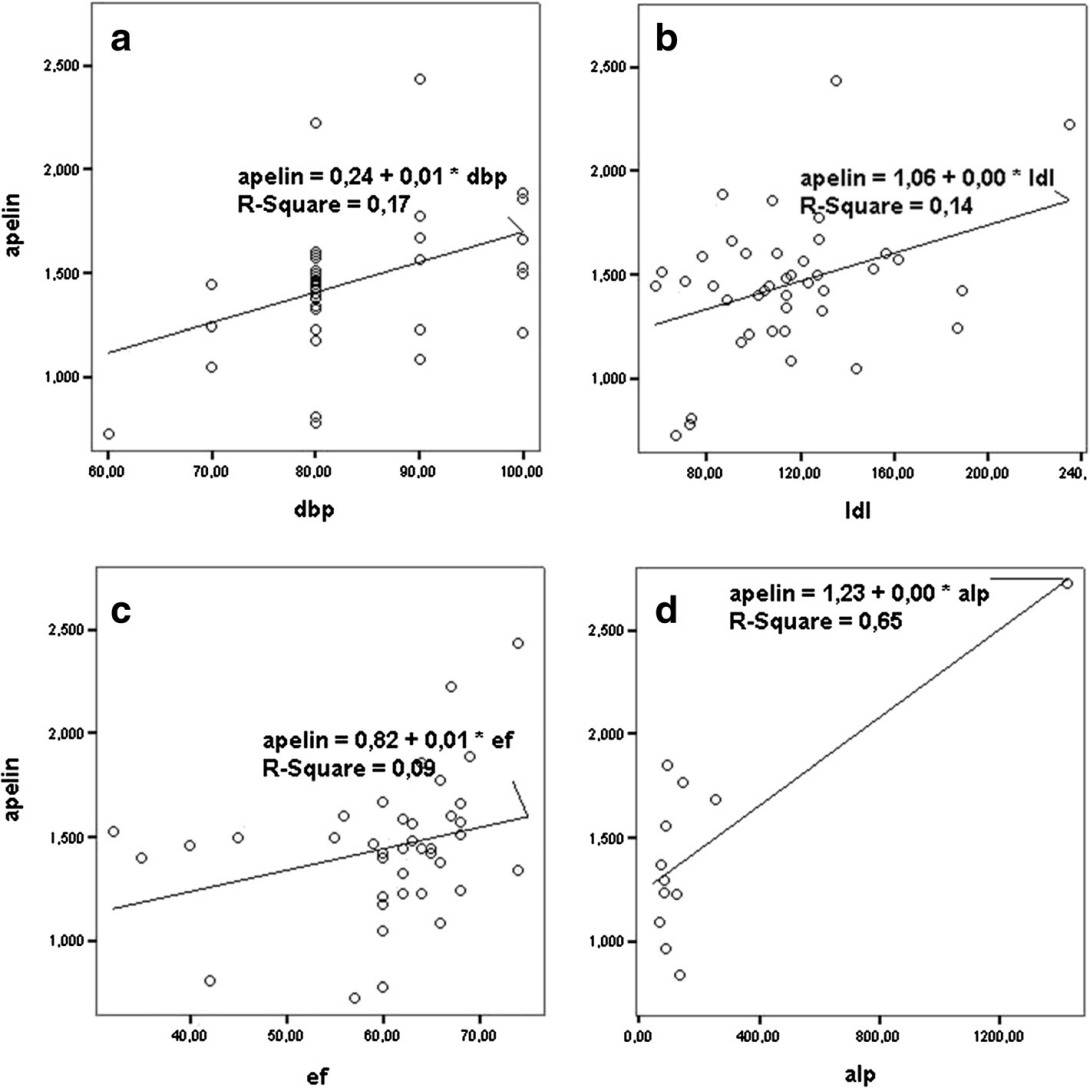
**The graphical presentation of parameters found in multivariated analysis to be correlated with apelin-36 levels in patients with (a,b,c) and without (d) hypertension.***dbp: Diastolic blood pressure; ldl: LDL cholesterol; ef: Ejection fraction; alp: Alkaline phosphatase.*

Linear regression model was applied with parameters found to be correlated with apelin-36 in univariate analysis besides gender, PD modality and primary kidney disease. Diastolic blood pressure, LDL-cholesterol, ALP and EF were found to be the independent determinants of apelin-36 levels (Table 4). When this analysis was repeated dividing the patients into two groups according to the presence of hypertension; diastolic blood pressure (B = 0.001, beta = 0.42, p = 0.002), LDL-cholesterol (B = 0.003, beta = 0.35, p = 0.021) and EF (B = 0.01, beta = 0.27, p = 0.049) remained as the determinants in hypertensive group; while ALP (B = 0.001, beta = 1.08, p = 0.009) was the only one in patients without hypertension (Figure 1).

**Table 4:** Results of linear regression analysis

	**B**	**Standard error**	**Beta**	**T**	**p**
**Constant**	-0.586	0.447		-1.311	0.196
**Diastolic blood pressure**	0.011	0.004	0.334	3.148	0.003
**Alkaline phosphatase**	0.001	0.000	0.487	4.630	0.000
**LDL-cholesterol**	0.003	0.001	0.256	2.438	0.019
**Ejection fraction**	0.011	0.004	0.265	2.499	0.016

## Discussion

It is very difficult to mention a standard normal level of apelin due to the presence of various forms of apelin (apelin-12, apelin-13, apelin-18, apelin-36) in the circulation. Besides this factor; different kits for measurement and the cross reaction between the types of apelin lead to various levels mentioned in the literature. Foldes et al. reported normal apelin levels as 89.8 ± 5.3 pg/ml [23]. Malyszko et al. reported that level as 84.0 ± 9.26 pg/ml in the general population and 49.16 ± 22.19 pg/ml in HD patients using the same assay (apelin-36 radioimmunoassay using commercially available kit from Phoenix Pharmaceuticals Inc., USA) with Foldes et al. [24]. Codognotta et al. found normal levels of apelin in the general population as 100 pg/ml [25]. Mean apelin-12 level was reported as 304 pg/ml in the normal population [26], 1.14 ng/ml in patients with stable angina pectoris [27]. El-Mesallamy [28] reported normal level of apelin-12 in the healthy population as 1.11 ng/ml.

The literature data about apelin levels in patients with ESRD is limited, so there is no cut-off value for it. But studies reported lower values in dialysis patients compared with the general population [24]. Apelin level was found to be lower in uremic patients with dilated cardiomyopathy than in nonuremic counterparts; which leads to the speculation that uremia decreases apelin levels irrespective of the degree of heart failure [25]. There is no data about the clearance of apelin during HD or PD in the literature. But it has been speculated that due to its molecular weight it is expected to be filtered through the glomerulus but not through low-flux hemodialysis membranes; so dialysis clearance can not be the reason of lower levels in HD patients [25]. We think that apelin may be cleared from the circulation in PD patients due to larger pore size of the peritoneal membrane.

The increased rate of CVD in uremic population is well known. One of the factors increasing cardiovascular risk in PD population is hypervolemia. In our study we found relationships between apelin-36 levels and diastolic blood pressure, left atrium diameter and EF all of which are related with volume status of the patients. Although patients with evident hypervolemia were excluded from the study; these relations which are present especially in the hypertensive group mark the relationship of apelin-36 with volume status. Positive correlation with EF may be an evidence for its positive inotropic action reported in previous studies [14,29,30]. Malyszko *et al.*[24] reported correlation between apelin levels and left ventricle end diastolic and end systolic diameters, biatrial diameters, right ventricle diameter, left ventricle posterior wall thickness and aorta diameter which are all indirect markers for volume status in HD patients.

We did not investigate directly the relationship between apelin-36 and body fluid status. But, when results of our study are considered together with literature data; it may be speculated that hypervolemia that is prevalent in dialysis population causes elevations in apelin levels to maintain compensatory diuresis, increased cardiac contractility and vasodilation. But there is not enough data to accept this theory as a fact.

The physiological effects of apelin mentioned before (positive inotropism, vasodilation, decreased blood pressure, and diuresis through effects on central nervous system) seem to antagonize deleterious effects of renin-angiotensin-aldosteron system (RAAS) [31]. It is clear that there is need for large scaled studies about the pathophysiological and therapeutic role of apelin in both uremic and non-uremic population.

Malyszko *et al.*[24] found apelin levels lower in HD patients with ischemic heart disease than those without ischemic heart disease. The levels were lower in patients with ischemic heart disease in our study also, but the difference was not statistically significant. This may be related with the small number of patients.

The strong correlation of apelin-36 with LDL and HDL-cholesterol is an interesting finding. Although apelin is secreted also from adipose tissue, this is not sufficient to explain the relationship. Malyszko *et al.*[24] found negative correlation between apelin and total cholesterol, LDL-cholesterol and triglyceride levels in HD patients. This difference from our study may be related with metabolic abnormalities like hyperglycemia, dyslipidemia and obesity that are more common in PD patients due to the glucose content of PD solutions. Moreover, Tasci *et al.*[25,26] found in their studies that apelin levels were lower in patients with high LDL-cholesterol in non-uremic population; and lowering LDL-cholesterol with life style changes and/or statins resulted in an increase in apelin levels. These different results between uremic and nonuremic population may be regarded as a clue for different lipid profile of uremic patients.

Another effect of apelin is diuresis resulting from inhibition of ADH release. Hus-Citharel *et al.*[16] reported significant diuresis after infusion of apelin-17 to rats. Lack of correlation between apelin-36 levels and daily urine output in our study may be due to blunted diuretic effect of apelin-36 in patients with renal failure who are supposed not to respond to decreased ADH levels.

It has been shown by studies that there may be a relationship between apelin levels and inflammation [32]. Malyszko et al. reported apelin level as correlated with intracellular adhesion molecule, adiponectin and the presence of coronary arery disease in patients with transplanted kidneys [33]. El-Shehaby et al. found a negative correlation of apelin levels with interleukin-6 and hsCRP levels [34]. Although the aim of our study was not to investigate apelin-inflammation relation; there was no correlation with hsCRP levels.

Apelin has been found as an independent predictor of bone mineral density in post- menopausal women [35]. Other studies showed apelin expression in osteoblasts, and reported that apelin increases osteoblast proliferation while suppressing apoptosis of these cells [19,20]. In another study, apelin has been reported to decrease osteoblastic differentiation of vascular smooth muscle cells and vascular calcification which are important in the pathogenesis of CVD in uremia [21]. The significant correlation of apelin-36 with ALP and PTH detected in our study gains meaning considering these literature data. Apelinergic system may be supposed to be active in the pathogenesis of bone mineral metabolism disorders in uremic patients.

## Conclusion

Apelinergic system has important roles in volume regulation, cardiovascular functions, lipid metabolism and bone mineral disorders in PD patients. Prospective studies with large population are required.

## Competing interests

The authors declared that they have no competing interests.

## Authors’ contributions

SK: Mainly planned and conducted the study; and wrote the paper. SO: Participated in the design of the study and performed statistical analysis. MG: Participated in the design of the study and preparation of the manuscript. AG: Organized the planning and analysis of the cardiological part of the study; and helped writing the manuscript. FB: Performed the biochemical analysis and helped statistical analysis. SY: Performed the biochemical analysis and helped statistical analysis. ZA: Participated in the design of the study and preparation of the manuscript. SU: Participated in the design of the study and preparation of the manuscript AS: Participated in the design of the study and preparation of the manuscript. HO: Organized the planning and analysis of the cardiological part of the study; and helped writing the manuscript. RK: Conceived of the study, and participated in its design and coordination and helped to draft the manuscript. All authors read and approved the final manuscript.

## Pre-publication history

The pre-publication history for this paper can be accessed here:

http://www.biomedcentral.com/1471-2369/15/18/prepub
